# Ginsenosides enhance P2X7-dependent cytokine secretion from LPS-primed rodent macrophages

**DOI:** 10.1007/s11302-023-09935-0

**Published:** 2023-04-14

**Authors:** Kshitija Dhuna, Ray Helliwell, Simone N. De Luca, Sarah J. Spencer, Leanne Stokes

**Affiliations:** 1 School of Health & Biomedical Sciences, RMIT University, Bundoora, VIC 2038 Australia; 2 School of Pharmacy, University of East Anglia, Norwich Research Park, Norwich, NR4 7TJ UK

**Keywords:** Ginsenoside, Cytokine, Macrophage, P2X7, ATP

## Abstract

The activation of P2X7 is a well-known stimulus for the NLRP3-caspase 1 inflammasome and subsequent rapid IL-1β secretion from monocytes and macrophages. Here we show that positive allosteric modulators of P2X7, ginsenosides, can enhance the release of three important cytokines, IL-1β, IL-6 and TNF-α from LPS-primed rodent macrophages using the J774 mouse macrophage cell line and primary rat peritoneal macrophages. We compared the immediate P2X7 responses in un-primed and LPS-primed macrophages and found no difference in calcium response amplitude or kinetics. These results suggest that under inflammatory conditions positive allosteric modulators are capable of increasing cytokine secretion at lower concentrations of ATP, thus boosting the initial pro-inflammatory signal. This may be important in the control of intracellular infections.

## Introduction

The P2X7 receptor is an ATP-gated ion channel which is well known to regulate inflammatory events in immune cells [1]. This includes pro-inflammatory cytokine secretion and the regulation of cell death pathways [2]. Cytokines are critical to the orchestration of the inflammatory response and the adaptive immune response under infectious conditions. Acute pro-inflammatory cytokines including interleukin-6 (IL-6), interleukin-1β (IL-1β) and tumour necrosis factor-α (TNF-α) play a crucial role in the activation of neutrophils, T cell differentiation and control of the fever response in response to infection [3]. We recently investigated how positive allosteric modulation of P2X7 with the ginsenoside compound K (CK) could affect the form of cell death induced by ATP in the J774 mouse macrophage cell line [4]. P2X7-dependent immediate signalling was enhanced in the presence of this positive allosteric modulator altering downstream signalling pathways ultimately changing the cell response [4]. In J774 mouse macrophages, stimulation with 500 µM ATP in the presence of the ginsenoside was able to change the cell death response to a caspase-3 dependent apoptotic form of death rather than following a lytic and inflammatory form of cell death [4].

In this short report we show that ginsenosides acting on P2X7 in rodent macrophages can potentiate P2X7 downstream signalling in LPS-primed cells and can significantly boost the secretion of pro-inflammatory cytokines from LPS-primed macrophages. This suggests that P2X7 activation in the presence of chemicals that can act as positive allosteric modulators would lead to an increase in pro-inflammatory signalling followed by apoptotic cell death and this may be important in inflammatory and infectious diseases.

## Methods

### Cell culture

The J774 mouse macrophage cell line was grown in RPMI 1640 medium containing 10% FBS (Sigma Aldrich, product code F2442) and 100 U/ml penicillin plus 100 µg/ml streptomycin (Thermo Fisher Scientific). Cells were maintained in a humidified incubator at 37˚C with 5% CO_2_. Peritoneal macrophages were extracted from adult male Wistar rats following CO_2_ asphyxiation. Animals were housed under standard laboratory conditions (12-h light cycle, 22 ˚C, 40–60% humidity with free access to water and standard rat chow) and were treated and culled in accordance with approval from the RMIT University Animal Ethics Committee and the Australian Code of Practice for the Care and Use of Animals for Scientific Procedures. PBS (5–10 ml) was used to flush the peritoneal cavity and the resulting cell suspension was centrifuged at 300x*g* for 5 min to pellet the cells. Peritoneal macrophages were resuspended in supplemented RPMI 1640 medium and used immediately in experiments.

### Calcium measurements

Cells were plated in poly-D-lysine coated 96-well plates the day before experiment at a density of 2 × 10^4^ cells/well. LPS priming was performed for 4 h before the experiment began using 100 ng/ml LPS (LPS from *Escherichia coli* 055:B5 Sigma Aldrich) in complete media. Cells were loaded with 2.25 µM fura-2AM in HBSS buffer containing 250 µM sulfinpyrazone (Sigma Aldrich). Following 40 min loading at 37 ˚C, buffer was removed and replaced with standard extracellular assay buffer (145 mM NaCl, 2 mM KCl, 2 mM CaCl_2_, 1 mM MgCl_2_, 13 mM glucose, 10 mM HEPES, pH 7.3). A Flexstation 3 plate reader (Molecular Devices) was used to record the Fura-2 ratio using excitation wavelengths of 340 nm and 380 nm and an emission wavelength of 520 nm.

### Cell viability

Cells were plated at 2.5 × 10^4^ cells/well and left for 24 h to adhere to the 96-well plate. Drug combinations were freshly prepared in media and were added for 24 h. During the last 4 h of incubation, Cell Titer 96 Aqueous solution (Promega) was added. The absorbance at 490 nm was measured using a Flexstation 3 plate reader.

### Caspase 3/7 measurements

Cells were plated in glass bottom 96-well plates and incubated with 1 µM NucView 488 caspase 3/7 substrate plus NucBlue in HBSS buffer containing 20 mM HEPES, pH 7.3. Following incubation for 40 min at room temperature, drugs were added and images were taken every 12 min using an ImageExpress (Molecular Devices). Recordings were performed at 37 ˚C. Two sites per well were used for data analysis (*n* = 500–700 cells per image).

### Cytokine assays

Cells were plated in 24-well plates at 3 × 10^5^ cells/well. Following LPS priming for 4 h (100 ng/ml), stimuli (ATP -/ + ginsenosides) were added for 30 min and plates were incubated at 37 ˚C. Cell-free supernatants were collected and frozen in Eppendorf tubes at -80 ˚C. Mouse/rat IL-6, TNF-α and IL-1β were assayed by ELISA using kits from Becton Dickinson and Abcam following the manufacturer’s instructions. Absorbance was measured at 450 nm using a Clariostar plate reader (BMG Labtech).

### Data and statistical analysis

Graphs were plotted and statistical analysis performed using GraphPad Prism version 6. For cell viability experiments data was expressed as percentage of control where control were cells treated with just media. Two-way ANOVA was performed with Tukey’s multiple comparison post-hoc test and significance taken as *P* < 0.05.

## Results

**Fig. 1:**
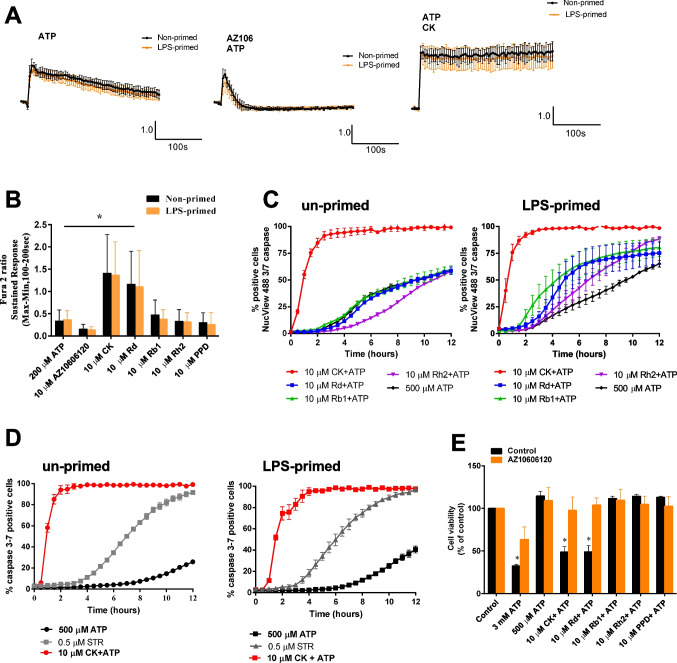
LPS priming does not affect positive allosteric modulation of P2X7-dependent responses in J774 mouse macrophages or primary rat peritoneal macrophages. **A** Intracellular calcium responses were measured in fura-2 loaded J774 mouse macrophages in the resting state (black traces) or 100 ng/ml LPS-primed (orange traces) for 4 h. ATP (200 µM) was automatically injected using Flexstation 3 fluidics in the absence or presence of ginsenoside CK (10 µM). Cells were pre-treated with AZ10606120 (10 µM) for 10 min before recording. Scale bars indicate fura-2 ratio change of 1.0 over time (100 s). **B** Quantification of the sustained phase (100–300 s) of the calcium responses from un-primed (black) or LPS-primed (orange) J774 mouse macrophages. **C** Measurement of caspase 3/7 activation over 12 h in un-primed and LPS-primed J774 mouse macrophages using NucView caspase 3/7 reagent and ImageExpress. Cells were exposed to 500 µM ATP in the absence (black) or presence of various ginsenosides; CK (red), Rd (blue), Rb1 (green or Rh2 (purple). Percentage caspase 3/7 positive cells was calculated using NucBlue stain to identify all cells. **D** Measurement of caspase 3/7 activation over 12 h in un-primed and LPS-primed primary rat peritoneal macrophages. Cells were exposed to 500 µM ATP in the absence (black) or presence of ginsenoside CK (red). Staurosporine (STR) 0.5 µM was used as a known inducer of cell death as a comparison. **E** Cell viability after 24 h incubation with stimuli in LPS-primed rat primary peritoneal macrophages using Cell Titer 96 Aqueous cell viability assay (MTS). Concentrations of ATP are indicated. 500 µM ATP was added in combination with ginsenosides tested. Data is presented as percentage of control using media treated cells as control. Orange bars indicate cells treated with AZ10606120 (10 µM). Data are mean ± S.E.M and two-way ANOVA using Tukey’s multiple comparison test was performed, * indicates *P* < 0.05

We first investigated whether LPS-primed J774 mouse macrophage cell line responded to P2X7 stimulation in the same way as un-primed J774 macrophages. This was important so we could understand whether LPS treatment for 4 hours would alter the functional expression of P2X7. Calcium responses to 500 µM ATP were similar in un-primed and LPS-treated J774 cells (Fig. 1A, B). The selective P2X7 antagonist AZ10606120 had the same effect on the ATP-induced calcium response, maintaining the initial transient elevation in [Ca^2+^]_i_ (due to the expression of P2Y receptors) but inhibiting the sustained phase of the [Ca^2+^]_i_ response (Fig. 1A, B). The inclusion of the positive modulator, ginsenoside CK (10 µM) dramatically enhanced the ATP-induced calcium response (Fig. 1A) as did ginsenoside Rd but not Rb1, Rh2 or the aglycone PPD (Fig. 1B). We next investigated caspase-3/7 activation in the 12 hours following stimulation of P2X7 in LPS-primed J774 mouse macrophage cell line. Caspase-3/7 activation was rapidly induced when cells were treated with a combination of ATP + CK in both un-primed and LPS primed J774 macrophages (Fig. 1C). In the presence of ginsenoside CK all cells became caspase 3/7 positive by 2 hours post-stimulation (Fig. 1C) however there was no difference in caspase 3/7 activation in the presence of Rd or Rb1 (Fig. 1C). In LPS-primed J774 macrophages the other ginsenosides Rd, Rb1 and Rh2 did accelerate the activation of caspase 3/7. In *ex vivo* primary rat peritoneal macrophages the combination of ATP + CK rapidly induced caspase 3/7 activation in both un-primed and LPS-primed cells (Fig. 1D).

**Fig. 2:**
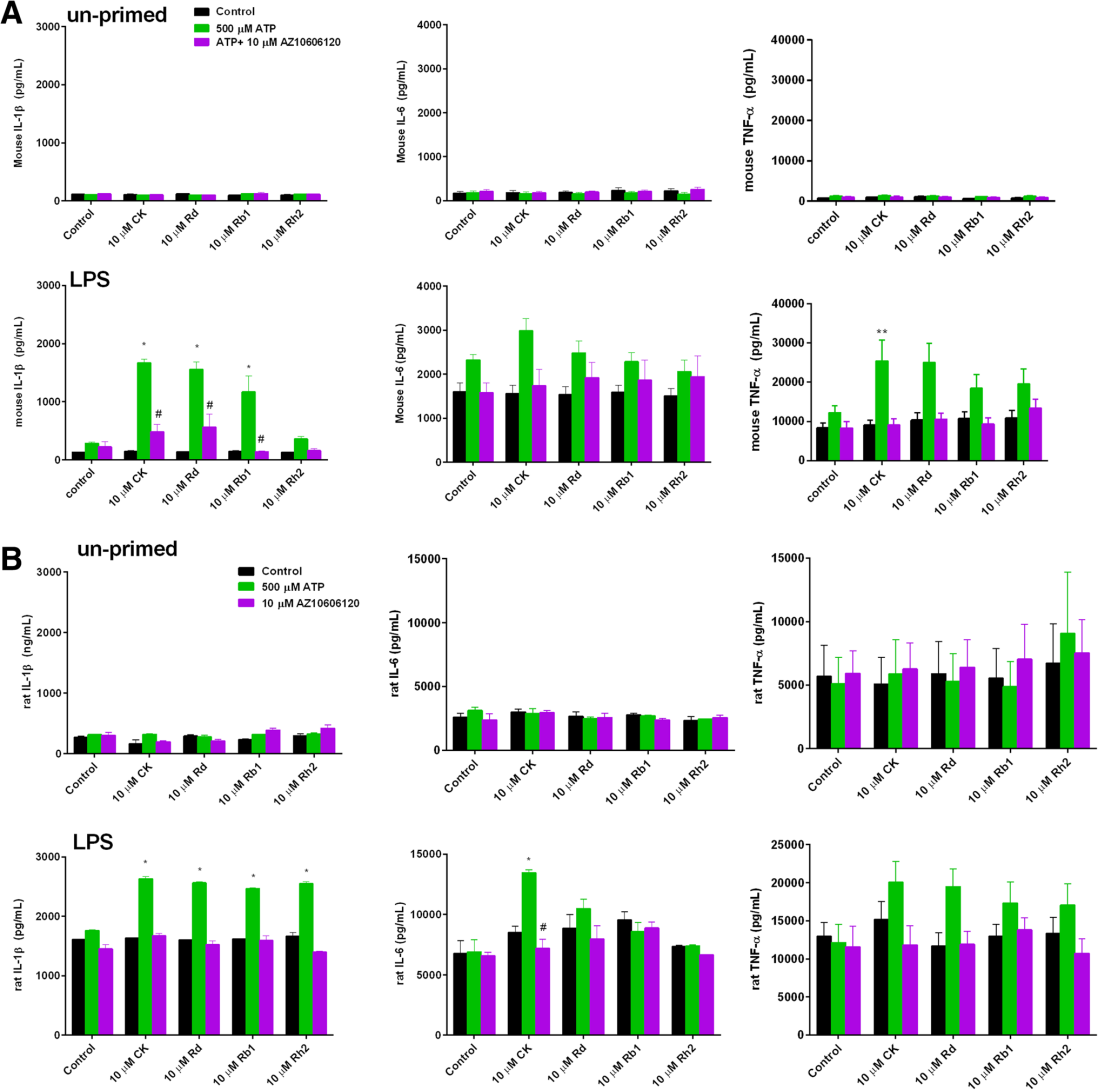
Ginsenosides can potentiate ATP-induced cytokine secretion from J774 mouse macrophages and rat peritoneal macrophages. **A** J774 mouse macrophage cell line, either un-primed or LPS-primed for 4 h, were treated with no stimuli (black bars), 500 µM ATP (green bars) or 500 µM ATP in the presence of 10 µM AZ10606120 (purple bars) and a variety of different ginsenosides as indicated. After 30 min cell-free supernatants were collected and analysed for IL-1β, IL-6 or TNF-α by ELISA. Data is from 3 independent experiments. **B** Ex vivo primary rat peritoneal macrophages were un-primed or LPS primed for 4 h, and treated with no stimuli (black bars), 500 µM ATP (green bars) or 500 µM ATP in the presence of 10 µM AZ10606120 (purple bars) and a variety of different ginsenosides as indicated. Data is averaged from 6 preparations of rat macrophages. Error bars indicate S.E.M. Data was analysed using two-way ANOVA with Tukey’s post hoc test and * indicates *P* < 0.05

We confirmed that the combination of ATP (500 µM) plus ginsenoside CK (10 µM) could induce cell death in primary rat peritoneal macrophages measured as a reduction in cell viability following a 24 hours incubation similar to our previous work [4, 5]. This reduction in cell viability was prevented by pre-incubation with AZ10606120 (Fig. 1E). Therefore, P2X7 responses are unchanged in LPS-primed cells and can be potentiated by ginsenoside CK altering immediate calcium influx and downstream caspase 3/7 activation. Under these LPS-primed conditions we next wanted to assess the effect of ginsenosides on P2X7-dependent cytokine secretion from J774 macrophages and *ex vivo* primary rat peritoneal macrophages. We measured IL-1β, IL-6 and TNF-α secretion from un-primed and LPS-primed J774 mouse macrophage cell line (Fig. 2A) and rat primary peritoneal macrophages (Fig. 2B). For these experiments we used a concentration of ATP that does not typically induce a cytokine secretion response, 500 µM. Ginsenoside treatment had the largest effect on IL-1β secretion from LPS-primed macrophages, both J774 mouse macrophage cell line and primary rat peritoneal macrophages, with ginsenosides CK, Rd and Rb1 all potentiating ATP-induced cytokine secretion in the 30-min timeframe (Fig. 2A, B). There was also significant potentiation of TNF-α secretion from LPS-primed J774 macrophages with ginsenoside CK (Fig. 2A). For the rat peritoneal macrophages, the overall picture was similar, however, the ginsenosides only significantly potentiated ATP-induced IL-1β secretion and CK potentiated IL-6 secretion (Fig. 2B). Altogether this data demonstrates that in the presence of a positive allosteric modulator of P2X7, micromolar concentrations of ATP can cause release of several pro-inflammatory cytokines from both mouse and rat macrophages with a similar pattern across both species.

## Discussion

Much of the work on P2X7 portrays the activation of this ligand-gated ion channel to be detrimental and likely to contribute to inflammatory diseases, as reviewed in [1, 6, 7]. However, there is a growing body of evidence that P2X7 activation can be beneficial in fighting infectious diseases [7, 8] building on earlier knowledge of anti-microbial killing mechanisms induced by P2X7 [9–12]. P2X7 is known to require high concentrations of ATP for activation and there has been much debate regarding how millimolar concentrations of ATP can be achieved and sustained in the extracellular space in order to effectively activate P2X7. In the case of infection, inflammation or trauma, ATP is known to be released from dying cells due to cell lysis and ATP can also be released via physiological processes from immune cells [13]. In the presence of a positive allosteric modulator, the concentration of ATP required to activate P2X7 can be reduced into the 50–500 µM range and this could be further exploited to help boost anti-microbial killing mechanisms [14]. Moreover, this idea has been tested using clemastine, a non-selective positive modulator of human P2X7 [15], in a zebrafish model of mycobacterial infection [16] with success.

Many studies use LPS as a priming agent to ensure expression of all components of the key inflammasome proteins (e.g. ASC, NLRP3 and caspase-1), although this is dispensable in some cell types such as monocytes [17]. P2X7 has been linked to IL-1β, TNF-α and IL-6 secretion from macrophages [18] and other cell types [19] although these latter two cytokines are measured less often. In this study we compared P2X7 dependent calcium influx in un-primed and LPS-primed J774 macrophages and found no difference in the amplitude of the calcium response suggesting P2X7 activation gives rise to a similar immediate response. Others have suggested that LPS can bind to the P2X7 C-terminus [20] and some have shown that LPS can inhibit the ion channel function of P2X7 although this used a higher concentration of LPS on recombinant human P2X7 [21]. In our study, priming with LPS did not significantly affect caspase-3 activation by the combination of ATP and ginsenoside CK, however, in LPS-primed J774 macrophages the other ginsenosides were also now effective at enhancing caspase-3 activation. Only CK and Rd could enhance ATP-mediated cell death in *ex vivo* rat peritoneal macrophages after 24 hours. Our major question regarding cytokine secretion was to determine whether this downstream signal would be enhanced given the alteration in intracellular signalling pathways (e.g. caspase-3 activation). Stimulation induced a robust inflammatory signal leading to IL-1β, TNF-α and IL-6 secretion from J774 macrophages and this was mirrored in primary rat peritoneal macrophages. This is the first investigation into the effects of ginsenosides on P2X7-mediated cytokine secretion however, our work is in line with previous literature on other positive allosteric modulators of P2X7. Polymyxin B has been shown to increase ATP-induced IL-1β secretion from J774 macrophages [22] and clemastine increased IL-1β secretion from LPS-primed human monocyte-derived macrophages [15].

A recent review article documents the good (angel) vs bad (devil) role of P2X7 in infectious models [7] and how the nature of the particular pathogen can play a role in determining this. For some infections inhibition or deletion of P2X7 is protective (e.g. sepsis, HIV) whereas in other infections, it appears to be deleterious to inhibit P2X7 (e.g. *Leishmania amazonensis*, *Toxoplasma gondii*). In these protozoan and helminthic infections it may be beneficial to enhance P2X7 responses, particularly in individuals carrying loss-of-function polymorphisms in P2X7. Certain single nucleotide polymorphisms have been shown to influence infection outcomes [23]. We recently showed that ginsenosides could potentiate ATP-mediated responses in the THP-1 human monocyte cell line which is known to carry the 1513A > C (rs3751143) polymorphism [24]. Therefore, we suggest that more work should be performed to determine whether boosting P2X7 responses in infection models is a useful therapeutic angle. The recent work from Douget et al. showing the beneficial effect of HEI3090, a small molecule positive modulator of mouse P2X7, on anti-tumour responses in combination with immunotherapy [25] suggests that the benefits of positive allosteric modulation of P2X7 may have multiple therapeutic benefits and is an exciting area for future research.

## Data Availability

Data is available upon reasonable request.

## Code availability

N/A.
